# Conservation, duplication, and loss of the Tor signaling pathway in the fungal kingdom

**DOI:** 10.1186/1471-2164-11-510

**Published:** 2010-09-23

**Authors:** Cecelia A Shertz, Robert J Bastidas, Wenjun Li, Joseph Heitman, Maria E Cardenas

**Affiliations:** 1 Department of Molecular Genetics and Microbiology, Duke University Medical Center, Durham, NC 27710, USA

## Abstract

**Background:**

The nutrient-sensing Tor pathway governs cell growth and is conserved in nearly all eukaryotic organisms from unicellular yeasts to multicellular organisms, including humans. Tor is the target of the immunosuppressive drug rapamycin, which in complex with the prolyl isomerase FKBP12 inhibits Tor functions. Rapamycin is a gold standard drug for organ transplant recipients that was approved by the FDA in 1999 and is finding additional clinical indications as a chemotherapeutic and antiproliferative agent. Capitalizing on the plethora of recently sequenced genomes we have conducted comparative genomic studies to annotate the Tor pathway throughout the fungal kingdom and related unicellular opisthokonts, including *Monosiga brevicollis*, *Salpingoeca rosetta*, and *Capsaspora owczarzaki*.

**Results:**

Interestingly, the Tor signaling cascade is absent in three microsporidian species with available genome sequences, the only known instance of a eukaryotic group lacking this conserved pathway. The microsporidia are obligate intracellular pathogens with highly reduced genomes, and we hypothesize that they lost the Tor pathway as they adapted and streamlined their genomes for intracellular growth in a nutrient-rich environment. Two *TOR *paralogs are present in several fungal species as a result of either a whole genome duplication or independent gene/segmental duplication events. One such event was identified in the amphibian pathogen *Batrachochytrium dendrobatidis*, a chytrid responsible for worldwide global amphibian declines and extinctions.

**Conclusions:**

The repeated independent duplications of the *TOR *gene in the fungal kingdom might reflect selective pressure acting upon this kinase that populates two proteinaceous complexes with different cellular roles. These comparative genomic analyses illustrate the evolutionary trajectory of a central nutrient-sensing cascade that enables diverse eukaryotic organisms to respond to their natural environments.

## Background

The nutrient-sensing target of rapamycin (Tor) pathway is highly conserved among eukaryotes and governs several essential cellular processes including protein synthesis, ribosome biogenesis, autophagy, and cytoskeletal organization [1-3]. In the fungal kingdom, the Tor pathway has been best studied in the budding yeast *Saccharomyces cerevisiae *[2-4], the fission yeast *Schizosaccharomyces pombe *[5,6], and the human pathogen *Candida albicans *[5-8]. However, little is known about Tor in basal fungal lineages, including the Zygomycota and Chytridiomycota.

In *S. cerevisiae *and *S. pombe*, two Tor paralogs form distinct complexes known as Tor Complex 1 (TORC1) and Tor Complex 2 (TORC2) [9-12], while in most other species, including humans, a single Tor protein can populate both complexes [11-14]. Interestingly, *S. cerevisiae *Tor2 can complement the loss of Tor1, but Tor1 cannot complement the loss of Tor2 [15,16]. Two Tor paralogs have also been identified in a metazoan, the silkworm *Bombyx mori *[17] and three Tor paralogs were identified in the trypanosomatid parasites *Leishmania major *[18] and the related species *Trypanosoma brucei *[19], the first reported Tor triumvirates.

**Figure 1:**
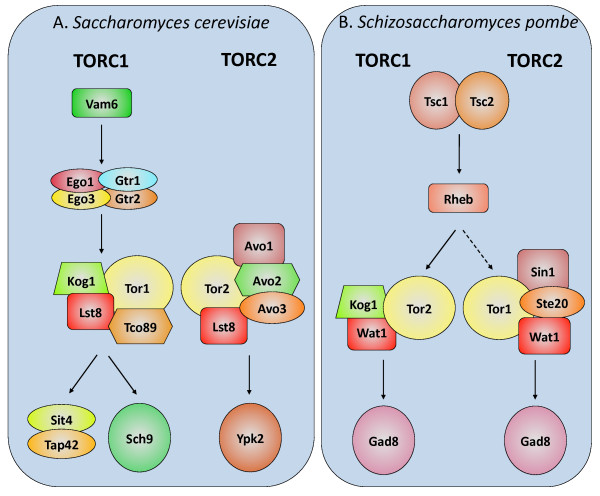
**The Tor pathway in the model fungi *Saccharomyces cerevisiae *and *Schizosaccharomyces pombe***. The Tor pathway components investigated in this study in *S. cerevisiae *(A) and *S. pombe *(B) are included in this figure. Functional homologs between the two species are indicated in the same shape and color. Sch9, Ypk1, and Gad8 are AGC kinases that are Tor- and PDK-regulated.

The ScTORC1 is sensitive to rapamycin and controls protein synthesis, mRNA synthesis and degradation, ribosome biogenesis, and autophagy. TORC2 is insensitive to rapamycin and is involved in the control of actin polarization and cell wall integrity [9,16]. TORC1 consists of Tor1 or Tor2, Kog1 [20], Tco89, and Lst8 [21], while TORC2 contains Tor2, Lst8, Avo1, Avo2, and Avo3 [22] (Figure 1A). Recently, the EGO-GTPase complex and its orthologs were shown to convey amino acid signals for TORC1 activation in yeast, insects, and mammals [23-26]. In *S. cerevisiae*, immediate effectors of TORC1 include the PP2A-like phosphatase Sit4 [27] and the AGC kinase Sch9 [28]. In *S. pombe*, Tor participates in other cellular functions including nutrient signaling [29], cell growth and differentiation [30], mitotic commitment [31], and sexual development [11] (Figure 1B).

**Figure 2:**
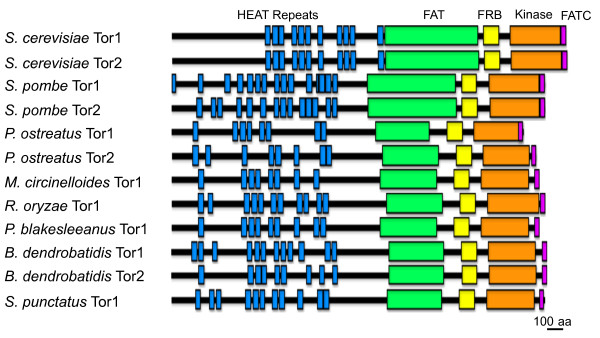
**Tor protein architecture**. Tor protein domain architecture is highly conserved throughout the fungal kingdom. The N-terminal HEAT repeats (blue), the FAT (green) and the FATC (purple) domains participate in protein-protein scaffolding thereby facilitating complex interactions. The FRB domain (yellow) is a highly conserved 100 amino acid sequence necessary for rapamycin interaction. The kinase domain (orange) phosphorylates protein substrates.

The structure of the Tor proteins is remarkably conserved (Figure 2) and features several domains for protein-protein interactions including N-terminal HEAT (Huntingtin, Elongation factor 3, PP2A A subunit, Tor) repeats [32], a FAT (FRAP, ATM, and TRRAP) domain [33,34], and an FRB (FK506-Rapamycin Binding) [35] domain. The kinase and the FATC (FAT domain at the C-terminus) [32,36] domains are present in the C-terminal region.

The Tor inhibitor rapamycin blocks cell proliferation and is currently used as an immunosuppressive drug for organ and tissue transplant recipients and a chemotherapy agent against a variety of solid cancers [37-40]. Rapamycin binds to the prolyl isomerase FKBP12 to form a protein-drug complex that then interacts with the Tor FRB domain in a ternary complex [41,42]. *S. cerevisiae *cells treated with rapamycin display phenotypes associated with nutrient depletion including G1 cell cycle arrest, cellular volume expansion, protein synthesis inhibition, glycogen accumulation, and autophagy [35,43].

Genetic analysis in *S. cerevisiae *characterizing rapamycin-resistant mutants led to the identification of FKBP12 as the intracellular receptor for rapamycin and defined Tor1 and Tor2 as the targets of the FKBP12-rapamycin complex [4]. Subsequent studies resulted in the identification and characterization of the *TOR1 *and *TOR2 *gene products [15,44] as well as the elucidation of the Tor signaling cascade [8,45-47]. These pioneering studies aided in the identification of the mammalian Tor ortholog and characterization of this highly conserved signaling cascade [8,48,49]. Remarkably, expression of the human FKBP12 ortholog in yeast *fpr1 *(the gene that encodes FKBP12) deletion mutants complements to restore rapamycin sensitivity, and hybrid Tor proteins consisting of the yeast N-terminal domain fused to the mammalian Tor kinase domain are also functional in yeast [50]. Thus, the Tor pathway has been functionally and structurally conserved from yeasts to humans over the billion years of evolution separating the two species from their last common ancestor.

We now know that the fungal and metazoan kingdoms are both within the opisthokont lineage of eukaryotes and are thus more closely related to each other than the vast majority of eukaryotic organisms [51,52]. Moreover, these two highly successful kingdoms shared a last common ancestor as recently as one billion years ago, much more recently than most eukaryotes. Recent initiatives, particularly the UNICORN project, have facilitated the sequencing of several opisthokonts related to the last common ancestor of the metazoans and fungi, providing an interesting window into the evolution of both fungi and animals [53-56]. Thus, studies on the evolutionary trajectory of the fungal kingdom in general, and of the Tor signaling cascade in particular, promise to reveal insights about how orthologous pathways function in the more complex milieu of multicellular metazoan organisms.

In this study, we have capitalized upon the wealth of available genomic information by annotating the Tor pathway in several fungal organisms in which this pathway has not been described. Our study included selected species in the major groups of the fungal kingdom with genome sequences available: the basidiomycete *Pleurotus ostreatus*; the Mucorales zygomycetes *Mucor circinelloides, Rhizopus oryzae*, and *Phycomyces blakesleeanus*; the chytridiomycetes *Spizellomyces punctatus *and *Batrachochytrium dendrobatidis*; the microsporidian species *Encephalitozoon cuniculi, Enterocytozoon bieneusi*, and *Nosema ceranae*; and the related non-fungal opisthokonts *Capsaspora owczarzaki, Salpingoeca rosetta*, and *Monosiga brevicollis*. Whereas the Tor pathway is conserved throughout the eukaryotes, strikingly, microsporidian species with their highly reduced and compacted genomes lack the entire Tor pathway. We have also investigated gene and genome duplication events that resulted in two Tor homologs in *S. cerevisiae*, *S. pombe, P. ostreatus*, and *B. dendrobatidis*, and the loss of a second Tor homolog following a whole genome duplication event in *R. oryzae*.

## Results and Discussion

### Conservation of the Tor signaling pathway molecular components

The Tor pathway is well conserved among nearly all eukaryotic species examined to date. Tor is essential for life and is the target of the potent drug rapamycin in fungi, humans, and other eukaryotic organisms [2,12,19,41]. Tor originated early during the eukaryotic radiation, as it is present in the basal eukaryotes *Giardia lamblia *[57], *L. major *[18] and *T. brucei *[19], and also in plants [58]. Here we focused on the Tor pathway in the fungal kingdom and other representative unicellular opisthokont species outside of the fungi. Several genes encoding Tor complex components, upstream regulators, and downstream effectors were identified in all major groups throughout the fungal kingdom (Figure 1, Table 1). Because we used BLASTp reciprocal best hits (RBH) to identify orthologs, in some cases the absence of an ortholog in our results may represent only a failure to detect it with this method. Further, functional homologs that are not sufficiently similar enough in sequence to be identified may be present. Overall, a high degree of pathway conservation is observed in the Tor signaling cascade throughout the fungal kingdom, with the exception of microsporidia in which all Tor pathway components are absent. In addition, several pathway components are conserved in the related unicellular opisthokonts *M. brevicollis, S. rosetta*, and *C. owczarzaki *(Table 2).

**Table 1:** The presence or absence of Tor pathway components in non-pathogenic and pathogenic fungal lineages

		Ascomycota		Basidiomycota	Zygomycota			Chytridiomycota		Microsporidia
		
		*Sc*	*Sp*	*Po*	*Mc*	*Ro*	*Pb*	*Bd*	*Spu*	spp.
		
TORC1	Tor1	+	+	+	+	+	+	+	+	-
	Kog1	+	+	+	+	+	+	+	+	-
	Tco89	+	-	-	-	-	-	-	-	-
	Lst8	+	+	+	+	+	+	+	+	-
TORC2	Tor2	+	+	+	-	-	-	+	-	-
	Lst8	+	+	+	+	+	+	+	+	-
	Avo1	+	+	+	+	+	+	+	+	-
	Avo2	+	-	-	+	+	+	+	+	-
	Avo3	+	+	+	+	+	+	+	+	-
	Bit61	+	-	-	-	-	-	-	-	-

Upstream	Tsc1	-	+	+	+	+	+	-	+	-
	Tsc2	-	+	+	+	+	+	+	+	-
	Rhb1	+	+	+	+	+	+	+	+	-
	Ego1	+	-	-	-	-	-	-	-	-
	Ego3	+	-	-	-	-	-	-	-	-
	Gtr1	+	+	+	+	+	+	+	+	-
	Gtr2	+	+	+	+	+	+	+	+	-
	Vam6	+	+	+	+	+	+	+	+	-

Downstream	Sit4	+	+	+	+	-	+	+	+	-
	Tap42	+	+	+	+	+	+	+	+	-
	Sch9	+	+	+	-	-	-	+	+	-
	Ypk2	+	+	-	-	+	-	-	-	-

The presence or absence of Tor pathway components is indicated by + or -, respectively. Abbreviations: *Sc *= *S. cerevisiae*, *Sp *= *S. pombe, Po *= *P. ostreatus, Mc *= *M. circinelloides, Ro *= *R. oryzae, Pb *= *P. blakesleeanus, Bd *= *B. dendrobatidis, Spu *= *S. punctatus*.

**Table 2:** The presence or absence of Tor pathway components in selected unicellular opisthokonts

		Choanoflagellatea		Filasterea
		
		*Mb*	*Sr*	*Co*
		
TORC1	Tor1	+	+	+
	Kog1	+	-	+
	Tco89	-	-	-
	Lst8	+	-	+
TORC2	Tor2	-	-	-
	Lst8	+	-	+
	Avo1	-	-	-
	Avo2	-	+	+
	Avo3	+	+	+
	Bit61	-	-	-

Upstream	Tsc1	-	-	-
	Tsc2	+	+	+
	Rhb1	-	-	-
	Ego1	-	-	-
	Ego3	-	-	-
	Gtr1	-	+	+
	Gtr2	+	+	+
	Vam6	-	+	+

Downstream	Sit4	+	-	+
	Tap42	-	+	+
	Sch9	-	-	+
	Ypk2	-	+	+

The presence of absence of Tor pathway components is indicated by + or -, respectively. Abbreviations: *Mb *= *M. brevicollis*, *Sr *= *S. rosetta*, *Co *= *C. owczarzaki*.

In *S. cerevisiae*, Tor1 and Tor2 interact with several proteins to form TORC1 and TORC2. TORC1 contains Tor1 or Tor2, Kog1, Tco89, and Lst8. Kog1 functions as a substrate-recruiting subunit in mammalian TORC1 (mTORC1) [13,59-61]. A gene encoding a putative Kog1 homolog was identified in all species included in this study, with the exception of the microsporidia and *S. rosetta *(Tables 1 and 2). Mutation of *TCO89 *results in hypersensitivity to rapamycin and affects cellular integrity in *S. cerevisiae *[21]. Tco89 is only found in *S. cerevisiae *and could have resulted from a specialization of the pathway exclusive to *Saccharomyces *species (Table 1). However, while a BLASTp search did not identify Tco89 homologs in other species, *S. pombe *has been reported to contain a functional homolog [GenBank:NP_588232] [12], and this suggests that functional homologs may exist in other fungal species as well. However, no homologs were identified when using *S. pombe *Tco89 as a query sequence. Lst8 binds to the Tor kinase domain in *S. cerevisiae *to stimulate catalytic activity [9] and a putative Lst8 homolog is conserved in most species analyzed except microsporidia and *S. rosetta *(Tables 1 and 2).

Additional BLASTp searches identified a single Tor homolog in the following species: *Lachancea thermotolerans *CBS6340 [GenBank:XP_002552336], *Pichia pastoris *GS115 [GenBank:XP_002491471], *Pichia stipitis *CBS6054 [GenBank:XP_001385651], *Debaryomyces hansenii *CBS767 [GenBank:XP_002770885]*, Yarrowia lipolytica *CLIB122 [GenBank:XP_505106], *Podospora anserina *strain S mat+ [GenBank:XP_001903968], *Chaetomium globosum *CBS148.51 [GenBank:XP_001226647], *Magnaporthe oryzae *70-15 [GenBank:XP_001414541], *Gibberella zeae *PH-1 [GenBank:XP_388309], *Aspergillus fumigatus *Af293 [GenBank:XP_755360], *Aspergillus flavus *NRRL3357 [GenBank:XP_002377897], *Neosartorya fischeri *NRRL181 [GenBank:XP_001260509], *Aspergillus terreus *NIH2624 [GenBank:XP_001213640], *Aspergillus nidulans *FGSCA4 [GenBank:XP_663586], *Aspergillus clavatus *NRRL1 [GenBank:XP_001275326], *Aspergillus oryzae *RIB40 [GenBank: XP_001826216], and *Aspergillus niger *CBS513.88 [GenBank:XP_001397781]. These ascomycetes were not included in further studies.

In yeast and mammals, TORC2 controls spatial aspects of growth. TORC2 includes Tor2, Avo1, Avo2, Avo3, and Bit61 [9,22]. Avo1 plays an essential role in actin cytoskeleton polarization [22] and is conserved throughout the organisms that were the focus of this study (Table 1) except in *S. rosetta *and *C. owczarzaki *(Table 2). However, due to high divergence of the *AVO1 *sequence amongst the known homologs, it is possible that the *S. rosetta *and *C. owczarzaki *homologs cannot be identified using the BLASTp algorithm. Avo2 is a nonessential substrate adaptor for TORC2 [22] and potential homologs were identified in most species studied, with the exceptions of *S. pombe*, *P. ostreatus*, the three microsporidian species (Table 1), and *M. brevicollis *(Table 2). In *S. cerevisiae*, Avo3 (also know as Tsc11) controls cytoskeletal dynamics [9,22], and homologs are conserved throughout the species examined (Tables 1 and 2). Bit61, a nonessential protein that associates with TORC2 [21], was only identified in *S. cerevisiae *(Tables 1 and 2).

Upstream regulators of Tor include Tsc1 and Tsc2 (Tuberous Sclerosis 1 and 2) and the GTPase Rheb (Ras homolog enhanced in brain). In *S. pombe *and other eukaryotes, Tsc1 and Tsc2 form a GTPase-activating complex that negatively regulates the action of Rheb to activate TORC1 [26,62,63]. Tsc1 and Tsc2 putative homologs were identified in most species in the study, with the exception of *S. cerevisiae *(Table 1); however, Tsc1 homologs were not identified in *B. dendrobatidis*, *S. rosetta*, or *C. owczarzaki *(Tables 1 and 2). In mammals and insects, four Rag GTPases (RagA-D) bind raptor (Kog1) to mediate TORC1 signaling in response to amino acids [24,26]. RagA and RagB are orthologs of *S. cerevisiae *Gtr1 whereas RagC and RagD are orthologs of *S. cerevisiae *Gtr2 [23]. Gtr1 and Gtr2 form a complex along with Ego1 and Ego3 known as EGOC/GSE [25], which is regulated by the GTP exchange factor Vam6 [23]. Interestingly we found presumptive Gtr1 and Gtr2 orthologs in species in this study with the exception of microsporidia (Table 1), and Gtr1 was not identified in *M. brevicollis *(Table 2). However, Ego1 and Ego3, which anchor Gtr1 and Gtr2 to endosomal and vacuolar membranes, are unique to *S. cerevisiae *and were not identified in the other species (Table 1). This suggests that the specific EGO complex architecture might be restricted to *Saccharomyces*, though it is possible that functional homologs may be present in other species.

In fungi, several downstream effectors are targets of Tor signaling. The PP2A phosphatase Sit4 and its regulatory subunit Tap42 regulate the expression of several TORC1-controlled genes and Gcn2-regulated translation [3,64,65]. A Sit4 homolog was identified in *R. oryzae *(Table 1), though a homolog of the regulatory subunit Tap42 was not (Table 1). In *S. rosetta*, a Sit4 homolog was not identified, though Tap42 was (Table 2). Both of these situations could indicate rewiring of the pathway, or alternatively these findings could be a result of insufficient similarity to detect with a RBH search method. Sch9, a member of the AGC kinase family and the functional homolog of the p70 S6 kinase, is a direct substrate of *S. cerevisiae *TORC1 involved in ribosome biogenesis [28,66]. Sch9 was not identified in RBH BLASTp searches with *S. cerevisiae *Sch9 in *M. circinelloides*, *P. blakesleeanus*, the microsporidia (Table 1), or *S. rosetta *(Table 2), but was identified in all other species. Putative homologs of Ypk2 kinase, a regulator of ceramide synthesis controlled by TORC2 signaling [67], were only identified in *S. cerevisiae*, *S. pombe*, (Table 1), *M. brevicollis, S. rosetta*, and *C. owczarzaki *(Table 2). Notably, the failure to detect many homologs in *M. brevicollis, S. rosetta*, and *C. owczarzaki *could be due to their evolutionary distance from the other organisms in the study and their gene divergence.

### Evolutionary conservation of Tor kinase protein structure

The Tor kinases are phosphatidylinositol kinase-related kinases (PIKKs) [68] and aside from their known protein kinase function also serve as evolutionary constrained scaffolds with several protein-protein interaction domains that mediate multi-protein complex formation [69]. Tor protein architecture was annotated using SMART [70,71] and we found that it has been maintained throughout the species of this study (Figure 2). N-terminal HEAT repeats are present in varying numbers and serve as scaffolding structures for protein-protein interactions [32,72,73]. Similarly, the FAT/toxic domain and the accompanying C-terminal FATC domain also participate in protein scaffolding and are present and conserved in all species subject to analysis. Over-expression of the FAT domain has a dominant negative effect on cell growth in yeast and this effect can be suppressed by over-expression of phospholipase C [33]. The kinase domain has similarities to both lipid and protein kinases, but it is a bona fide protein kinase. The FRB (FKBP12-rapamycin binding) domain is a highly conserved 100 amino acid region of Tor with several residues that are known to be required for binding the FKBP12-rapamycin drug complex [42].

**Figure 3:**
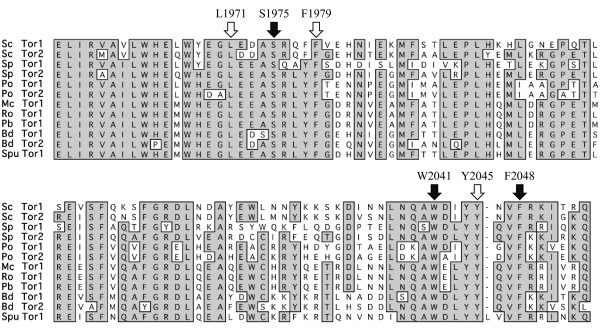
**The highly conserved FRB domain of Tor**. Residues L1971, F1979 and Y2045 are involved in phosphatidic acid binding in mTOR (open arrows). Mutation of S1975 confers rapamycin resistance in mammalian cells, *Candida albicans*, *Cryptococcus neoformans*, and *Saccharomyces cerevisiae*. Residues W2041 and F2048 are required for interaction with rapamycin. All of these amino acid residues are conserved in the species examined in this study. Abbreviations: Sc = *Saccharomyces cerevisiae*, Sp = *Schizosaccharomyces pombe*, Po = *Pleurotus ostreatus*, Mc = *Mucor circinelloides*, Ro = *Rhizopus oryzae*, Pb = *Phycomyces blakesleeanus*, Bd = *Batrachochytrium dendrobatidis*, Spu = *Spizellomyces punctatus*.

In addition, the FRB domain contains several residues necessary for both phosphatidic acid binding and rapamycin interaction [74,75]. In mammals, phosphatidic acid binding to the FRB region may promote the assembly of mTOR complexes. It has been proposed that the potent effect of the Tor inhibitor rapamycin may be due to direct competition for overlapping phosphatidic acid binding sites within the FRB domain [76]. Mutation of the conserved amino acid residues S1975, W2041, or F2048 in the FRB domain confers rapamycin resistance in *S. cerevisiae *[4,42,77]. Strikingly, the corresponding amino acid residues are conserved in all species examined (Figure 3). In mTOR, phosphatidic acid binding is disrupted by mutations of L2031, F2309, and Y2105 [75], corresponding to *S. cerevisiae *Tor2 L1971, F1979, and Y2045, respectively, all of which are conserved in the studied fungal species (Figure 3).

### Duplication of Tor in the fungal kingdom

Approximately 100 million years ago, the *S. cerevisiae *ancestor underwent a whole genome duplication (WGD) event, and species that descend from this evolutionary event retained duplicated subsets of genes. *S. cerevisiae *retained approximately 8% of duplicated genes [78,79], while other fungi in this lineage have maintained varying numbers of gene duplicates, such as *Candida glabrata *in which a smaller number (~2%) of these pairs are maintained [80]. Interestingly, several of these species, including *S. cerevisiae, S. paradoxus, S. mikatae, S. kudriavzevii, S. bayanus, S. castellii*, and *C. glabrata *have retained two *TOR *paralogs, whereas species outside of the WGD clade, including *Kluyveromyces lactis *and *Ashbya gossypii*, have only a single *TOR *gene.

The fates of duplicated gene paralogs can be explained through the duplication, degeneration, and complementation (DDC) model of gene duplication [81]. In species with a single Tor, this protein populates both TORC1 and TORC2, each of which has distinct functions. Following duplication, each paralog likely sub-functionalized to carry out some, but not all, of its previous functions so that between the two paralogs, each process is performed. In *S. cerevisiae*, for example, Tor1 exclusively functions within TORC1, while Tor2 preferentially populates TORC2 but can also function in TORC1. *S. pombe *has two Tor paralogs as a result of an independent segmental gene duplication event, and each paralog has distinct roles not necessarily equivalent to those of *S. cerevisiae *Tor1 and Tor2. SpTor1 is not essential, whereas SpTor2 is essential for growth [11,29,82].

We hypothesize that similar models could apply in species encoding two Tor homologs resulting from independent gene duplication events such as *P. ostreatus *and *B. dendrobatidis*. Based on their level of identity with *S. cerevisiae *Tor homologs, the *B. dendrobatidis *genes BDEG_08293 and BDEG_05727 have been designated *TOR1 *and *TOR2*, respectively. However, these gene names are not necessarily based on functional similarity with ScTor1 and ScTor2 but rather indicate only that there are two *TOR *homologs in *B. dendrobatidis*. A similar naming challenge is evident in *S. pombe*, where the functions of SpTor1 and SpTor2 are not equivalent with those of ScTor1 and ScTor2.

**Figure 4:**
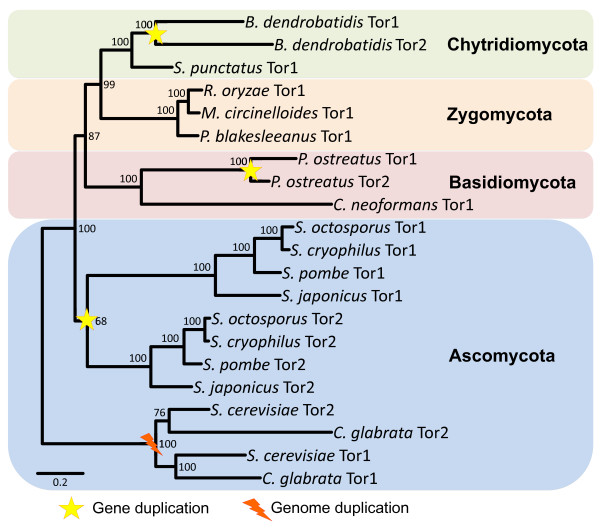
***TOR *gene duplication events**. Several independent segmental gene duplications or whole genome duplication events have occurred throughout the fungal kingdom resulting in multiple Tor homologs. A whole genome duplication occurred in the *Saccharomyces *budding yeast lineage prior to the speciation of the *sensu stricto*, *sensu lato*, and related *Saccharomycotina *species. An independent gene duplication event occurred in the *Schizosaccharomyces *lineage, resulting in 2 Tor homologs in four *Schizosaccharomyces *fission yeast species. Independent gene duplication events also occurred in *Batrachochytrium dendrobatidis *and the edible mushroom *Pleurotus ostreatus*. Numbers at nodes are bootstrap percentages representing 500 replicates.

**Figure 5:**
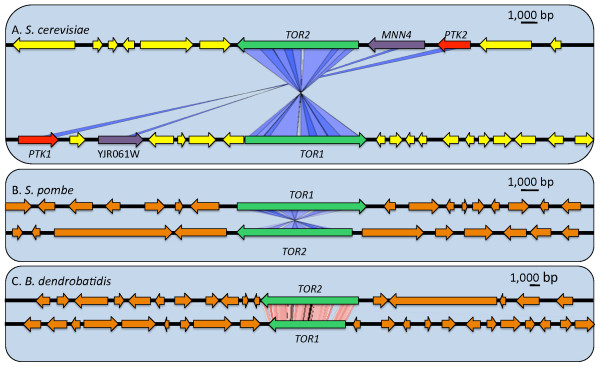
**Synteny analysis of *TOR *paralogs**. Synteny analysis supports the hypothesis that the Tor paralogs in *Saccharomyces cerevisiae *(A) resulted from a whole genome duplication event, while in *Schizosaccharomyces pombe *(B) and *Batrachochytrium dendrobatidis *(C) two Tor paralogs result from independent segmental gene duplication events, and there is no syntenic conservation in the surrounding sequence. Red lines indicate syntenic genes oriented in the same direction whereas blue lines indicate syntenic genes oriented in the opposite direction (i.e., + strand and - strand).

While several species, such as those in the *Saccharomyces *clade, have two Tor paralogs resulting from ancient WGD events, other species have acquired a second paralog through independent segmental gene duplication events, including *B. dendrobatidis *and several fission yeast species closely related to *S. pombe *(Figure 4). Segmental gene duplications have played a major role in the evolution of species and can result in the duplication of single genes or large blocks of genes [83]. In *S. cerevisiae*, the duplication blocks with *TOR *include three paralogous gene pairs that have been retained from the ancestral WGD event: *PTK1*/*PTK2*, *TOR1*/*TOR2*, and *MNN4*/YJR061W [78] (Figure 5A). Further, *C. glabrata*, an ascomycete closely related to *S. cerevisiae *that diverged following the WGD, has also maintained a second *TOR *paralog and synteny is apparent in the region flanking the *TOR1 *and *TOR2 *genes, though one must extend the analysis to >40 kb from the *TOR *genes to detect it (See additional file 1: Supplemental figure 1). However, because *C. glabrata *and *S. cerevisiae *have retained different subsets of genes from the ancestral WGD, the duplicated genes surrounding *TOR *in these species are not homologs of one another. Further, *C. glabrata *has maintained fewer of the synteny blocks found in *S. cerevisiae*, and the block containing the *TOR *genes (Block 42) is not conserved in *C. glabrata *[80].

In contrast, genomic regions surrounding the Tor paralogs in *S. pombe *(Figure 5B) and *B. dendrobatidis *(Figure 5C) are not syntenically conserved. That is, the duplicated genes are not flanked by other duplicated gene pairs, and the two *TOR *paralogs likely resulted from independent segmental gene duplication events. Further, there are no indications of WGDs in these species. In the Schizosaccharomycotina, the *TOR *gene duplication occurred prior to speciation as evidenced by the fact that *S. pombe, S. octosporus, S. japonicus*, and *S. cryophilus *have each retained a second Tor paralog; the Tor orthologs across these four species are more closely related to each other than to those from other species (Figure 4). No syntenic conservation between the genomic regions containing *TOR *paralogs was detected in any species of the *Schizosaccharomyces *group, supporting the occurrence of an independent segmental gene duplication in their common ancestor (see additional file 2: supplemental Figure 2).

The Taphrinomycotina is a monophyletic taxon of the Ascomycota containing the Schizosaccharomycetes, Taphrinomycetes, Neolectomycetes, and Pneumocystidomycetes. Genomes are only available for species within the Schizosaccharomycetes (*S. pombe, S. octosporus, S. japonicus*, and *S. cryophilus*) and the Taphrinomycetes (*Pneumocystis carinii*). While the studied *Schizosaccharomyces *spp. contain two Tor homologs, a single homolog was identified in *P. carinii *with tBLASTx using the highly conserved *S. cerevisiae *FRB domain of Tor1 based on sequence data produced by the *Pneumocystis *Genome Project (funded by the NIH NIAID) that was obtained from http://pgp.cchmc.org at the time of publication. Based on the phylogenetic placement of these two groups [84] and the available genomic information, we hypothesize that the segmental *TOR *duplication is unique to the *Schizosaccharomyces *spp. and occurred following their divergence from the common ancestor of the Taphrinomycota, as *P. carinii*, the other species within this taxon with available genome data, appears to contain a single Tor homolog.

Interestingly, the zygomycete *R. oryzae *underwent a whole genome duplication event and despite retaining 12% of the resulting duplicated genes [85] compared to 8% retained genes in a separate WGD event in *S. cerevisiae *[78,79], two Tor paralogs have not been retained; only a single Tor homolog was identified. Currently, the Zygomycota is the only group studied without an identified species containing two Tor homologs; however, there are not enough available genomes to make a definitive conclusion in this group. Other species with independent *TOR *duplications likely remain to be identified in all of the major fungal lineages, and as more genome sequences become available this will be addressed.

During this study, we identified three additional species that possibly have two *TOR *paralogs: *M. brevicollis*, *Thecamonas trahens*, and *Allomyces macrogynus*, species that are included in the Origins of Multicellularity Project [86]. While analysis is limited because of the state of the *M. brevicollis *(choanoflagellate) genome, it appears that an additional bonafide Tor protein is encoded with the conserved Tor protein architecture. *Thecamonas trahens *is a unicellular apusomonad that also contains two TOR paralogs. Interestingly, *A. macrogynus *is a fungus that may contain a second paralog; however, it appears that the FRB domain overlaps with the kinase domain. This may represent a case in which *TOR *was duplicated but is in the process of losing one of the copies. These cases are a springboard for future studies involving the duplication of *TOR *within and beyond the fungal kingdom.

### Loss of the Tor signaling pathway in microsporidia

While components of the Tor signaling cascade are conserved in eukaryotes from yeasts to humans, the microsporidia, a group of highly specialized obligate intracellular pathogens completely lack all of the pathway components investigated in this study. *E. cuniculi *has previously been noted to lack the Tor kinase [87], and we found that all pathway components including Tor were missing not only in *E. cuniculi *but also in its microsporidian relatives *E. bieneusi *and *N. ceranae *(Table 2). Microsporidian genomes are highly compacted and show a marked reduced gene content. The genome of the human pathogen *E. cuniculi *has 1997 genes distributed throughout 11 chromosomes in a 2.9 Mb genome [88], less than half the number of protein-coding genes found in *Escherichia coli*. For comparison, the genome of the human pathogen *E. bieneusi *genome is ~6 Mb with 3,804 genes [89] and the honeybee pathogen *N. ceranae *genome is ~7.9 Mb with 2,614 predicted genes [90]. Genome reduction has been well studied in intracellular bacteria: during the specialization process from a free-living to intracellular lifestyle, massive gene loss can occur [91-95]. We hypothesize that as a consequence of their specialization to an obligate intracellular lifestyle, the need for nutrient sensing was relaxed and these obligate intracellular microsporidia acquired nutrients from within the host cytoplasm. Thus, they no longer required the Tor pathway, which is dedicated to sensing changes in the nutrient composite of the environmental milieu. However, *E. cuniculi *has retained some of the genes encoding signaling pathways involved in nutrient sensing in other species, including homologs of *S. cerevisiae *Ras1 and Ras2 (data not shown).

Interestingly, *P. carinii *is an obligate pathogen with a reduced genome estimated to be ~8 Mb [96], which unlike microsporidia is not obligately intracellular. A single Tor homolog was identified [*Pneumocystis *Genome Project:cap3_it1_grp346_contig490] using the *S. cerevisiae *Tor1 FRB domain in tBLASTx, supporting our hypothesis that the microsporidia have streamlined their genomes as they adopted an obligate intracellular life cycle. *P. carinii *likely requires Tor because it must survive in a metabolically active form outside of host cells, undergoing cell division in the extracellular milieu of the infected host lung.

Many microsporidian genomes have undergone extreme genome reduction and are among the eukaryotes with the smallest genomes. In addition to fewer genes than other species (1997 in *E. cuniculi *compared to 6607 in *S. cerevisiae*), *E. cuniculi *has shorter genes, small intergenic distances, and very few introns [88,97]. This species (and to an even greater extent, *E. bieneusi*) lacks several genes that are necessary for biosynthetic pathways and the tricarboxylic acid cycle in free-living organisms [88,89,98]. Remarkably, *E. cuniculi *must acquire ATP from its host through a series of ATP transporters anciently acquired from *Rickettsia*- or *Chlamydia*-like bacteria [99], and this is an example of how *E. cuniculi*, and possibly other microsporidia, can hijack mechanisms of other intracellular bacteria, fungi, or the host to survive in and adapt to their highly specialized intracellular lifestyle.

## Conclusions

The Tor pathway is highly conserved with some exceptions among fungi, including several pathogens. Similarly, the molecular structural organization of the Tor kinases has been remarkably conserved as well as the presumed ability to bind the FKBP12-rapamycin complex. This feature, combined with the essential nature of the rapamycin-sensitive TORC1 pathway for cell growth, should enable the development of rapamycin-based strategies for antifungal therapies. Duplications of the Tor protein occurred in most fungal groups examined, resulting from either independent segmental gene duplication events or a WGD event. The maintenance of two Tor homologs can be explained through the DDC model of gene duplication, in which the paralogs subfunctionalize and together support all of the functions of the pre-duplicated protein. In addition, species outside of the fungal kingdom with two or more *TOR *homologs include the metazoan *B. mori*, the apusomonad protozoan *T. trahens*, the trypanosome protozoans *L. major *and *T. brucei*, and possibly the choanoflagellate *M. brevicollis*. This suggests that the Tor gene has been independently duplicated multiple times since the last common ancestor of the Opisthokonta and Excavata lineages. The Tor pathway, as well as the Tor protein itself, is highly conserved in eukaryotes, so the observation that three microsporidian species are missing the entire pathway is striking. It would appear that the specialized obligate intracellular life cycle of microsporidia allows for the acquisition of nutrients from the host cell, obviating a requirement for this central nutrient-sensing pathway that is essential in all known cases of other eukaryotes for axenic growth.

## Methods

### Identification of pathway component homologs

Tor homologs were identified using the highly conserved 100 amino acid FRB domain of *S. cerevisiae *Tor1 in BLASTp reciprocal best-hit searches between the specific species database and the *Saccharomyces *Genome Database (SGD). All queries for other Tor pathway components were retrieved from SGD and homologs were identified by BLASTp reciprocal best-hit searches, with the exception of Tsc1 and Tsc2. Tsc1 and Tsc2 protein query sequences were retrieved from the *S. pombe *GeneDB, and homologs were identified using BLASTp searches between the *S. pombe *genome database and the species database. Species identification numbers are listed in Supplemental Table 1. Protein architecture of each homolog was elucidated with Simple Modular Architecture Research Tool (SMART) analysis [70,71].

Accession numbers are provided for putative Tor pathway components (see Additional file 3, supplemental Table 1 for the accession numbers of putative Tor pathway components) from the following public genomic databases that were used to carry out BLAST: *S. cerevisiae*, SGD, http://www.yeastgenome.org; *S. pombe*, GeneDB, http://www.genedb.org/genedb/pombe/index.jsp; *P. ostreatus*, JGI, http://genome.jgi-psf.org/PleosPC15_1/PleosPC15_1.home.html; *M. circinelloides*, JGI and the Mucor Genome Project, http://genome.jgi-psf.org/Mucci1/Mucci1.home.html; *R. oryzae*, Broad Institute, http://www.broadinstitute.org/annotation/genome/rhizopus_oryzae/MultiHome.html; *P. blakesleeanus*, JGI, http://genome.jgi-psf.org/Phybl1/Phybl1.home.html; *B. dendrobatidis*, Broad Institute, http://www.broadinstitute.org/annotation/genome/batrachochytrium_dendrobatidis/MultiHome.html; *S. punctatus*, Broad Institute and the UNICORN initiative, http://www.broadinstitute.org/annotation/genome/multicellularity_project/MultiHome.html; *M. brevicollis*, JGI, http://genome.jgi-psf.org/Monbr1/Monbr1.home.html; *S. rosetta*, Broad Institute and the UNICORN initiative, http://www.broadinstitute.org/annotation/genome/multicellularity_project/MultiHome.html; *C. owczarzaki*, Broad Institute and the UNICORN initiative, http://www.broadinstitute.org/annotation/genome/multicellularity_project/MultiHome.html; *E. cuniculi, E. bieneusi*, and *N. ceranae*, NCBI, http://www.ncbi.nlm.nih.gov/sutils/genom_table.cgi?organism=fungi.

### Phylogenetic analysis of Tor

The ProtTest program [100] was used to select an appropriate model to construct the phylogenetic relationship of the Tor proteins of the studied species. For this study, the best model for phylogenetic construction of the Tor protein using maximum likelihood (ML) was JTT+I+G+F [101]. The ML tree was generated using PhyML with the JTT+I+G+F mode with 500 bootstrap replicates [102,103]. The Tor amino acid sequences from all species were aligned with CLUSTAL W [104].

### Synteny analysis of *TOR *paralogs

Tor homologs in fungal species were identified with BLAST [105] using *TOR1 *and *TOR2 *of *S. cerevisiae *as query sequences. The *TOR *sequence and flanking regions (up to 100 kb) were extracted from corresponding contigs or chromosomal sequences. To analyze the synteny of *TOR *and flanking regions, we constructed a database of one *TOR *and flanking region sequence and used the other *TOR *homolog and flanking region sequence to perform tBLASTx searches in the database (BLOSUM62 matrix; E-value = 1e-3; gap cost: existence 11, extension 1; and low complexity regions filter). The tBLASTx results were parsed and formatted using an in-house Perl script (available upon request). The parsed and formatted tBLASTx results were then imported into Artemis software [106] for synteny analysis. We employed a cutoff value of 100 bp to filter short non-significant matches between query and database sequences.

## Authors' contributions

CAS carried out the homolog searches and phylogenetic analysis and drafted the manuscript. RJB performed homolog searches and participated in the design of the study. WL performed the synteny and phylogenetic analysis and helped draft the manuscript. JH and MEC conceived of the study, participated in its design and helped to draft the manuscript. All authors read and approved the final manuscript.

## Supplementary Material

Additional file 1 **supplemental Figure 1 - Syntenic conservation of genomic area surrounding *TOR1 *and *TOR2 *in *Candida glabrata***. The top bar represents *C. glabrata *chromosome F and the bottom bar represents *C. glabrata *chromosome K. On chromosome F, the first five genes correspond to CAGL0F00110g, CAGL0F00121g, CAGL0F00143g, CAGL0F00154g, and CAGL0F00165g. Red lines indicate syntenic genes oriented in the same direction whereas blue lines indicate syntenic genes oriented in the opposite direction (i.e., + strand and - strand). Because *S. cerevisiae *retained 8% of its duplicated genes and *C. glabrata *retained only 2%, we had to use a larger window to detect syntenic gene pairs. *C. glabrata *has retained the other *S. cerevisiae *homologs (*PTK1*/*PTK2 *and *MNN4*/YJR061W) in the Tor block in duplicate, but they are located in separate blocks on *C. glabrata *chromosome 8, unlinked to *TOR1 *(chromosome 6) or *TOR2 *(chromosome 11). Click here for file

Additional file 2 **supplemental figure 2 - There is no syntenic conservation in *Schizosaccharomyces *species surrounding the *TOR *genomic regions**. Red lines indicate syntenic genes oriented in the same direction whereas blue lines indicate syntenic genes oriented in the opposite direction (i.e., + strand and - strand). No syntenic conservation was observed in the separate species, further supporting our hypothesis of an independent segmental gene duplication in the *Schizosaccharomyces *common ancestor. Click here for file

Additional file 3 **supplemental Table 1 - Accession numbers of putative Tor pathway components**. Putative Tor pathway components were assigned using reciprocal best hit BLAST matches with the following public databases: *S. cerevisiae*, SGD, http://www.yeastgenome.org; *S. pombe*, GeneDB, http://www.genedb.org/genedb/pombe/index.jsp; *P. ostreatus*, JGI, http://genome.jgi-psf.org/PleosPC15_1/PleosPC15_1.home.html; *M. circinelloides*, JGI and the Mucor Genome Project, http://genome.jgi-psf.org/Mucci1/Mucci1.home.html; *R. oryzae*, Broad Institute, http://www.broadinstitute.org/annotation/genome/rhizopus_oryzae/MultiHome.html; *P. blakesleeanus*, JGI, http://genome.jgi-psf.org/Phybl1/Phybl1.home.html; *B. dendrobatidis*, Broad Institute, http://www.broadinstitute.org/annotation/genome/batrachochytrium_dendrobatidis/MultiHome.html; *S. punctatus*, Broad Institute and the UNICORN initiative, http://www.broadinstitute.org/annotation/genome/multicellularity_project/MultiHome.html; *M. brevicollis*, JGI, http://genome.jgi-psf.org/Monbr1/Monbr1.home.html; *S. rosetta*, Broad Institute and the UNICORN initiative, http://www.broadinstitute.org/annotation/genome/multicellularity_project/MultiHome.html; *C. owczarzaki*, Broad Institute and the UNICORN initiative, http://www.broadinstitute.org/annotation/genome/multicellularity_project/MultiHome.html; *E. cuniculi, E. bieneusi*, and *N. ceranae*, NCBI, http://www.ncbi.nlm.nih.gov/sutils/genom_table.cgi?organism=fungi. Click here for file
