# Supplementary figures and images for: Conservation, duplication, and loss of the Tor signaling pathway in the fungal kingdom
Source: BMC Genomics. 2010 Sep 23;11:510. doi: 10.1186/1471-2164-11-510 (PMC2997006; doi:10.1186/1471-2164-11-510)

## Slide 1
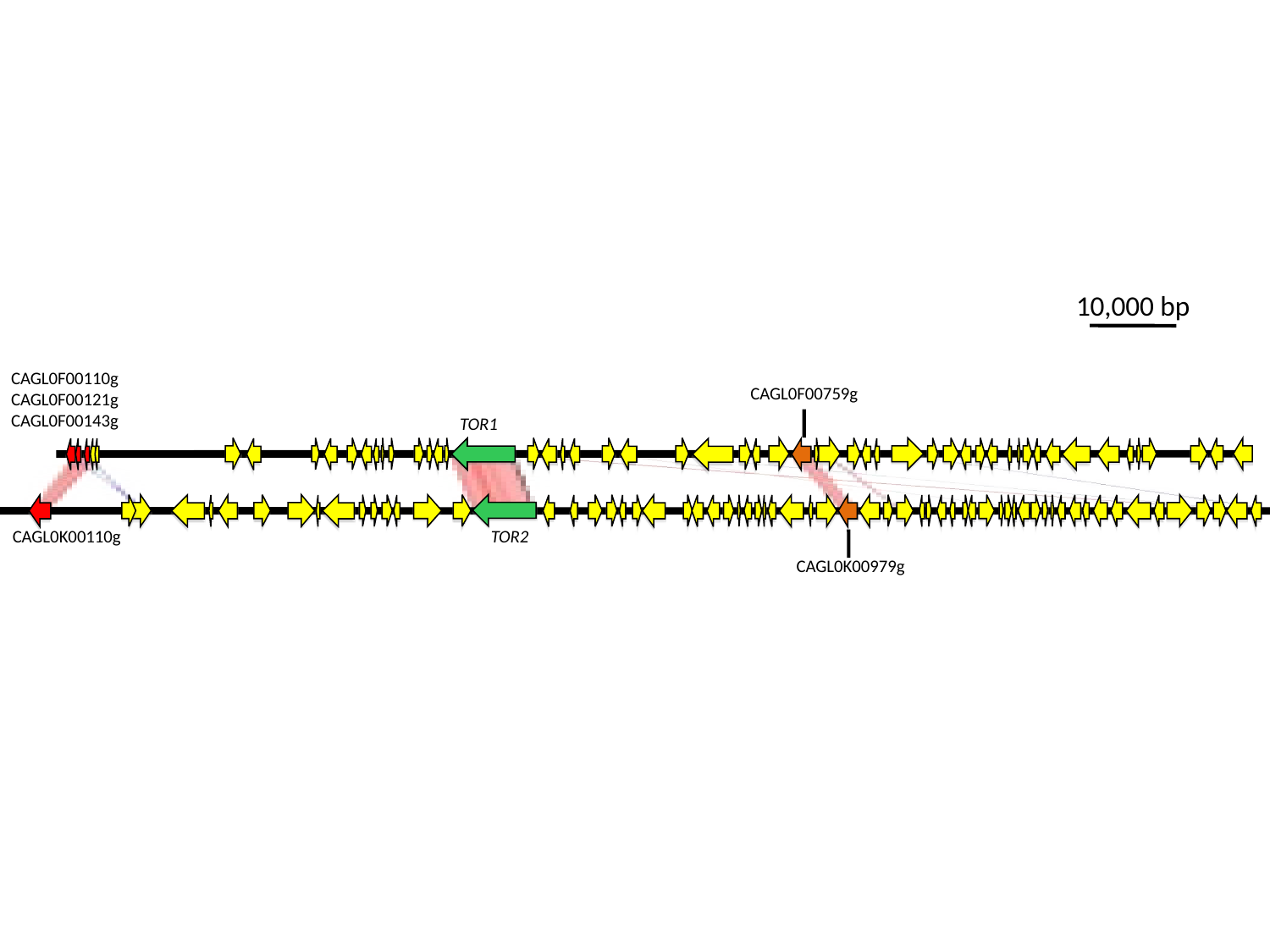

10,000 bp
CAGL0F00110g
CAGL0F00121g
CAGL0F00143g
CAGL0F00759g
TOR1
CAGL0K00110g
TOR2
CAGL0K00979g

Supplement: Additional file 1 — supplemental Figure 1 - Syntenic conservation of genomic area surrounding TOR1 and TOR2 in Candida glabrata. The top bar represents C. glabrata chromosome F and the bottom bar represents C. glabrata chromosome K. On chromosome F, the first five genes correspond to CAGL0F00110g, CAGL0F00121g, CAGL0F00143g, CAGL0F00154g, and CAGL0F00165g. Red lines indicate syntenic genes oriented in the same direction whereas blue lines indicate syntenic genes oriented in the opposite direction (i.e., + strand and - strand). Because S. cerevisiae retained 8% of its duplicated genes and C. glabrata retained only 2%, we had to use a larger window to detect syntenic gene pairs. C. glabrata has retained the other S. cerevisiae homologs (PTK1/PTK2 and MNN4/YJR061W) in the Tor block in duplicate, but they are located in separate blocks on C. glabrata chromosome 8, unlinked to TOR1 (chromosome 6) or TOR2 (chromosome 11). [file 1471-2164-11-510-S1.PPT]

## Slide 1
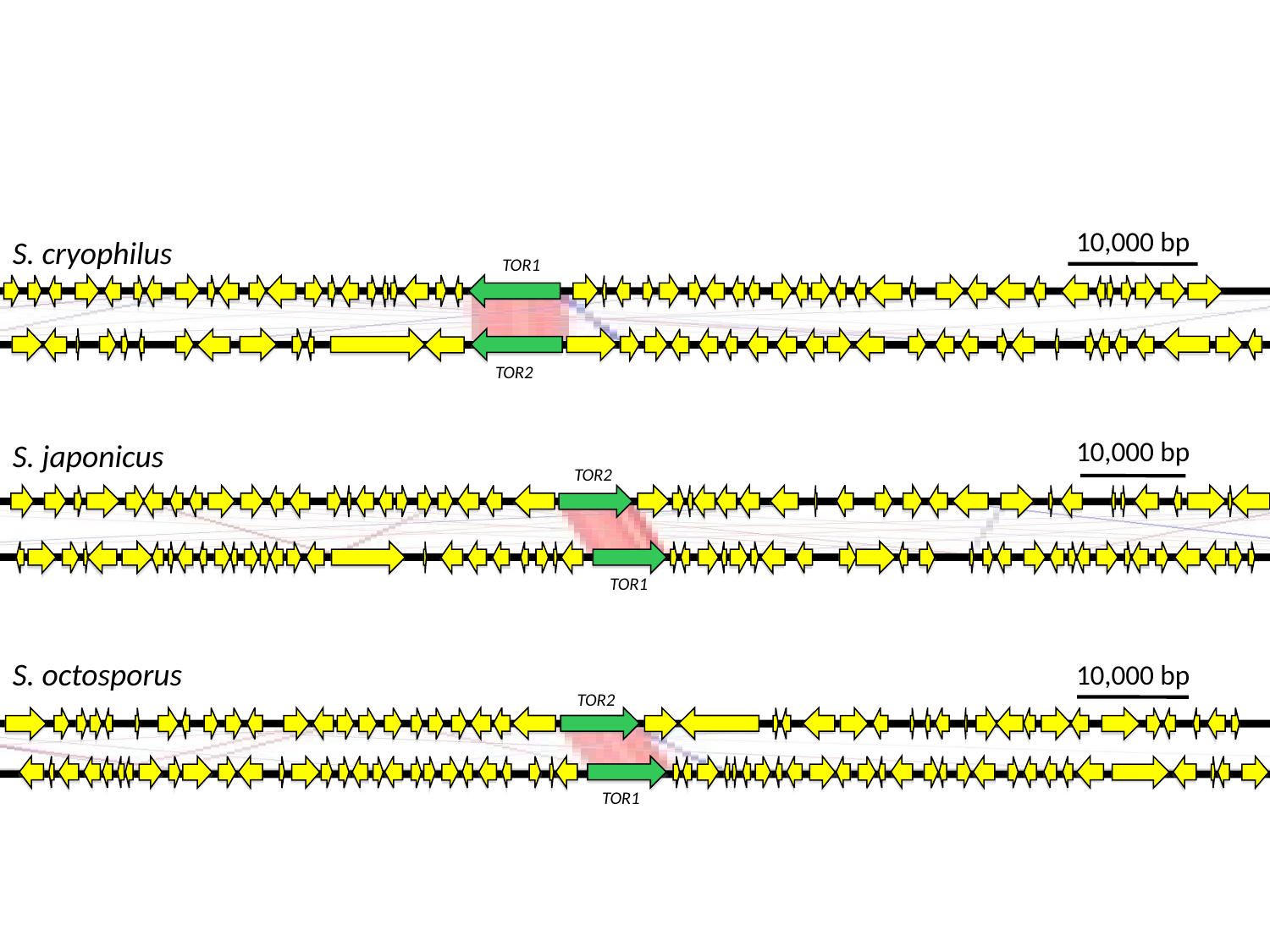

10,000 bp
S. cryophilus
TOR1
TOR2
10,000 bp
S. japonicus
TOR2
TOR1
S. octosporus
10,000 bp
TOR2
TOR1

Supplement: Additional file 2 — supplemental figure 2 - There is no syntenic conservation in Schizosaccharomyces species surrounding the TOR genomic regions. Red lines indicate syntenic genes oriented in the same direction whereas blue lines indicate syntenic genes oriented in the opposite direction (i.e., + strand and - strand). No syntenic conservation was observed in the separate species, further supporting our hypothesis of an independent segmental gene duplication in the Schizosaccharomyces common ancestor. [file 1471-2164-11-510-S2.PPT]
